# Effect of single‐ versus double‐layer uterine closure during caesarean section on postmenstrual spotting (2Close): multicentre, double‐blind, randomised controlled superiority trial

**DOI:** 10.1111/1471-0528.16472

**Published:** 2020-10-25

**Authors:** SI Stegwee, LF van der Voet, AJ Ben, RA de Leeuw, PM van de Ven, RG Duijnhoven, MY Bongers, CB Lambalk, CJM de Groot, JAF Huirne, for the 2Close study group, Dimitri NM Papatsonis, Eva Pajkrt, Wouter JK Hehenkamp, Angèle LM Oei, Mireille N Bekker, Daniela H Schippers, Huib AAM van Vliet, Lucet van der Voet, Nico WE Schuitemaker, Majoie Hemelaar, WM (Marchien) van Baal, Anjoke JM Huisjes, Wouter J Meijer, CAH (Ineke) Janssen, Wietske Hermes, AH (Hanneke) Feitsma, Hugo WF van Eijndhoven, Robbert JP Rijnders, Marieke Sueters, HCJ (Liesbeth) Scheepers, Judith OEH van Laar, Elisabeth MA Boormans, Paul JM van Kesteren, Celine M Radder, Esther Hink, Kitty Kapiteijn, Karin de Boer, Mesrure Kaplan, Erik van Beek, LHM (Marloes) de Vleeschouwer, Harry Visser, Judith E Bosmans, Mohamed El Alili, Josje Langenveld

**Affiliations:** ^1^ Department of Obstetrics and Gynaecology Amsterdam Reproduction & Development Amsterdam UMC Vrije Universiteit Amsterdam Amsterdam the Netherlands; ^2^ Department of Obstetrics and Gynaecology Deventer Hospital Deventer the Netherlands; ^3^ Department of Health Sciences Amsterdam Public Health Faculty of Science Vrije Universiteit Amsterdam Amsterdam the Netherlands; ^4^ Department of Obstetrics and Gynaecology Amsterdam Reproduction & Development Amsterdam UMC Universiteit van Amsterdam Amsterdam the Netherlands; ^5^ Department of Epidemiology and Biostatistics Amsterdam UMC Vrije Universiteit Amsterdam Amsterdam the Netherlands; ^6^ Department of Obstetrics and Gynaecology Máxima Medical Centre Research School Grow Maastricht University Veldhoven the Netherlands

**Keywords:** Caesarean section, double layer, niche, postmenstrual spotting, single layer, uterine closure technique

## Abstract

**Objective:**

To evaluate whether double‐layer uterine closure after a first caesarean section (CS) is superior compared with single‐layer uterine closure in terms of postmenstrual spotting and niche development in the uterine caesarean scar.

**Design:**

Multicentre, double‐blind, randomised controlled superiority trial.

**Setting:**

Thirty‐two hospitals in the Netherlands.

**Population:**

A total of 2292 women aged ≥18 years undergoing a first CS were randomly assigned to each procedure (1:1): 1144 women were assigned to single‐layer uterine closure and 1148 women were assigned to double‐layer uterine closure.

**Methods:**

Single‐layer unlocked closure and double‐layer unlocked closure, with the second layer imbricating the first.

**Main outcome measures:**

Number of days with postmenstrual spotting during one menstrual cycle 9 months after CS. Secondary outcomes: perioperative and menstrual characteristics; transvaginal ultrasound measurements.

**Results:**

A total of 774 (67.7%) women from the single‐layer group and 770 (67.1%) women from the double‐layer group were evaluable for the primary outcome, as a result of drop‐out and amenorrhoea. The mean number of postmenstrual spotting days was 1.33 (bootstrapped 95% CI 1.12–1.54) after single‐layer closure and 1.26 (bootstrapped 95% CI 1.07–1.45) after double‐layer closure (adjusted mean difference −0.07, 95% CI −0.37 to 0.22, *P* = 0.810). The operative time was 3.9 minutes longer (95% CI 3.0–4.9 minutes, *P* < 0.001) and niche prevalence was 4.7% higher (95% CI 0.7–8.7%, *P* = 0.022) after double‐layer closure.

**Conclusions:**

The superiority of double‐layer closure compared with single‐layer closure in terms of postmenstrual spotting after a first CS was not shown. Long‐term obstetric follow‐up of our trial is needed to assess whether uterine caesarean closure guidelines should be adapted.

**Tweetable abstract:**

Double‐layer uterine closure is not superior for postmenstrual spotting after a first caesarean; single‐layer closure performs slightly better on other outcomes.

## Tweetable abstract

Double‐layer uterine closure is not superior for postmenstrual spotting after a first caesarean; single‐layer closure performs slightly better on other outcomes.

## Introduction

Globally, caesarean section (CS) rates have risen from 12.1% (2000) to 21.1% (2015).^1^ Parallel with this rise, an increased incidence of CS‐related maternal morbidity is expected.^2^ Besides severe but fortunately rare obstetric events, more prevalent long‐term maternal symptoms after CS are dysmenorrhea and abnormal uterine bleeding.^2^, ^3^, ^4^

Some long‐term maternal symptoms are considered to be related to the appearance of the uterine scar, and more specifically to a niche in the caesarean scar as a surrogate marker.^4^, ^5^, ^6^ A niche is defined as an indentation in the myometrium of ≥2 mm in depth and is detectable by transvaginal ultrasound (TVUS), preferably with contrast in order to limit false negatives.^7^ When contrast is used, niche prevalence varies between 56 and 84%.^8^ Complications in subsequent pregnancies, including uterine rupture and placenta accreta spectrum disorders, are associated with thin residual myometrium (i.e. uterine myometrial tissue overlying the niche).^9^ ^,^ ^10^ A frequently reported gynaecological symptom is postmenstrual spotting (25%), which is associated with both thin residual myometrium and the presence and size of a niche.^4^, ^5^, ^6^ This chronic symptom is considered to be a major contributor to impaired maternal quality of life, sexuality and social interaction in the long term.^11^

The aetiology of niche development remains unknown. Contributing factors for niche development are multiple CSs and a thinning of the lower uterine segment during labour, but also involve surgery‐related factors, including the level of hysterotomy.^12^, ^13^, ^14^ Single‐ versus double‐layer closure of the uterine incision as a surgery‐related risk factor remains a topic of debate. Double‐layer closure is associated with a lower prevalence of large niches and thicker residual myometrium, but clinical outcomes are lacking.^15^, ^16^

No uniform evidence‐based guideline exists on uterine closure technique after CS: the American College of Obstetricians and Gynecologists (ACOG) has no specific clinical guideline. In the National Institute for Health and Care Excellence (NICE) guideline, double‐layer closure is advocated as the ‘effectiveness and safety of single‐layer closure is uncertain’.^17^ The Enhanced Recovery After Surgery (ERAS) Society also recommends double‐layer closure based on a presumed lower uterine rupture rate.^18^ No other maternal outcomes are taken into account.

We aimed to study the superiority of double‐layer unlocked uterine closure on postmenstrual spotting 9 months after a first CS, compared with single‐layer unlocked uterine closure.

## Methods

### Study design

We performed this multicentre, double‐blind, randomised controlled trial at the maternity unit of 32 university, teaching and district hospitals in the Netherlands, within the Dutch Consortium for Healthcare Evaluation and Research in Obstetrics and Gynaecology. The study protocol was published before data analysis began.^19^ The study was approved by the Institutional Review Board (IRB) of Amsterdam UMC, location VUmc (reg. no. 2015.462), and by the boards of all participating hospitals before enrolment started. No substantial changes were made to the protocol after the commencement of the trial.

### Participants

We recruited potential participants when a first CS took place. They were recruited when a CS was scheduled, at the outpatient clinic or delivery ward before labour started or at the delivery ward when in labour with adequate analgesia. We considered patients eligible for the study if they underwent a first CS, were over 18 years of age and had a good comprehension of Dutch or English language. We applied the following exclusion criteria: inadequate possibility for counselling; previous major uterine surgery; women with known causes of menstrual disorders; placenta increta or percreta during the current pregnancy; or three or more fetuses in the current pregnancy. All women who met our inclusion criteria and who were willing to participate provided written informed consent before the CS.

### Randomisation and masking

After informed consent had been signed and a CS was indicated, participants were randomly allocated to receive single‐layer or double‐layer closure of the uterine incision. Randomisation was performed with a 1:1 ratio using alea 2.2, a web‐based interface displaying the allocation from a computer‐generated randomisation sequence. Randomisation was stratified by hospital and timing of CS (i.e. prelabour or intrapartum), and within each stratum block randomisation was used where block sizes of two and four were alternated. The enrolment of participants and actual randomisation was carried out by research nurses, midwives, residents or gynaecologists only after the decision for a CS was made. The allocated closure technique was passed on to the performing surgeon who was thus not blinded to the allocated method. The researcher that performed the statistical analyses was not blinded to the group assignment. Participants, sonographers and supervising statisticians were blinded for the closure technique used.

### Procedures

In both arms, women underwent a CS with standard mode of uterotomy and correct approximation of the cutting edges. Double‐layer closure of the uterine incision was performed using unlocked continuous multifilament sutures for both layers, with a large portion of the myometrium and the endometrium included in the first layer. The second layer was a continuous running suture that imbricated the first layer, including serosal and myometrial tissue. Participating surgeons were instructed by a mandatory online instruction video prior to participation (Figure S1). Single‐layer closure of the uterine incision was performed using unlocked continuous multifilament sutures. As a result of inconclusive evidence with respect to endometrial‐saving technique, surgeons could choose to either include or exclude the endometrium in the single‐layer group, which was registered. We used data from the operative report for secondary end points.

At baseline, and at 3 and 9 months of follow‐up, we sent participants a digital questionnaire. The response to these questionnaires was used to assess the baseline characteristics, the primary outcome and several secondary outcomes.

**Figure 1:**
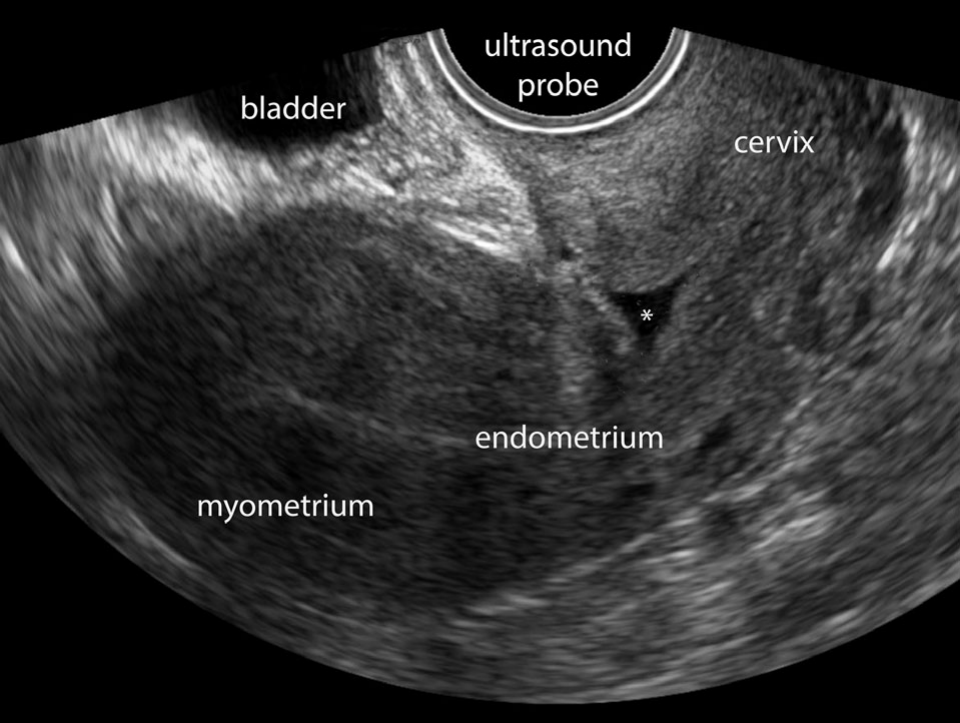
Niche after caesarean section on transvaginal ultrasound, without the use of contrast. A niche is visible, indicated with an asterisk *, in the caesarean uterine scar.

All participants were invited for sonographic uterine scar evaluation using TVUS at 3 months after CS. A structural assessment of the scar, including the detection and measurement of a niche, was performed according to the niche measurements guideline, endorsed by European experts (Figure 1).^7^ Sonographers followed a mandatory online e‐learning module in order to standardise the evaluation of the uterine scar (Figure S2). Validation of the first ultrasounds in each hospital was performed by the senior investigator of the study (JH). When TVUS was inconclusive or no niche was visible, patients were asked whether a contrast‐enhanced (either with saline or gel) ultrasound could be made, as sonohysterography is considered to be the gold standard for niche detection.^5^, ^7^, ^20^ We chose this approach to limit costs and participant discomfort. Sonographic evaluation at 3 months was chosen because the niche tends to change in appearance over time and this seemed the most appropriate and achievable time point.^8^, ^21^

### Outcomes

The primary outcome measure was number of days of postmenstrual spotting during a single menstrual cycle at 9 months after CS. This time frame was chosen to reduce the number of participants who were still breastfeeding but also to minimise the number of participants with a subsequent pregnancy. We defined postmenstrual spotting as brownish discharge for more than 2 days at the end of menstruation, with a total duration (menstruation and spotting) of more than 7 days, or intermenstrual blood loss that starts after the end of the menstruation.^4^ The number of days with spotting were reported by the patients through a digital questionnaire, including a calendar in which women could record their daily blood loss. Amenorrhoeic women were not evaluable for the primary outcome and were omitted from the analyses for this outcome.

Secondary outcome measures were prespecified in the study protocol and are summarised in Table S1.^19^ Serious adverse events were reported to the IRB through yearly line listing. A cost‐effectiveness analysis was performed alongside the trial from a societal perspective and will be published elsewhere. Additionally, we will evaluate the long‐term reproductive outcomes at 3 years of follow‐up in a separate publication.

### Sample size and statistical analysis

Based on previous research, a reduction in the mean number of postmenstrual spotting days per month from 3.50 (standard deviation, SD 3.44) to 3.00 was considered clinically relevant. An independent sample Student’s *t*‐test at a two‐sided significance level of 5% required a total of 1488 women to achieve 80% power. This number was increased by 35% to 2290 to account for women with amenorrhoea, dropping out or lost to follow‐up.

Demographic and preoperative data are presented as *n* (%) for categorical variables and mean (SD) or median (interquartile range, IQR) for continuous variables.

The primary outcome was first compared between study arms using a Mann–Whitney *U*‐test followed by analysis using quantile regression, in which we adjusted for the stratification variable ‘timing of CS’ and the baseline variables upon which the study arms differed. Adjustment for the stratification variable ‘hospital performing the CS’ was not possible because of the small number of participants in some hospitals. The model‐based difference in median is reported as effect size. In a secondary analysis, we used bootstrapping to determine the percentile‐based confidence interval and *P* value for the difference in means (10 000 samples).

Dichotomous outcomes were analysed using generalised linear models with (adjusted) relative risks (RRs) and (adjusted) absolute risk differences (RDs) reported as effect size. Continuous outcomes were analysed using linear regression with (adjusted) mean difference as effect size. Skewed continuous outcomes were first log‐transformed, after which we obtained the (adjusted) geometric mean ratio. When residuals were not normally distributed, we used quantile regression and calculated the (adjusted) difference in medians. We reported effect sizes for double‐layer relative to single‐layer uterine closure with 95% confidence intervals (95% CIs). Changes in repeatedly measured normally distributed outcomes (SF‐36 summary scores) were compared between arms using linear mixed models or the Mann–Whitney *U*‐test on change scores when the assumption of normality was violated.

Subgroups were compared on primary outcome by testing the interaction between subgroup variable and study arm in the appropriate regression model.

Within the single‐layer closure group, we additionally compared the primary outcome and secondary menstrual and sonographic outcomes when the endometrium was excluded and when the endometrium was included in the suture.

Quantile regression and linear mixed‐model analyses were performed in stata 14 (STATA, College Station, TX, USA). spss 24 (IBM, Armonk, NY, USA) was used for all other analyses. Primary analysis was performed according to the intention‐to‐treat (ITT) principle and additional per‐protocol analyses were performed excluding women who received a different uterine closure technique than they were allocated to. The analysis and presentation of results was carried out according to CONSORT (Consolidated Standards of Reporting Trials) guidelines. No interim analyses were performed. Missing data were assumed to be missing at random.

As this study was approved by the IRB and judged as an extremely low‐risk trial, no data monitoring committee was installed. This trial is registered in the Netherlands National Trial Register (NTR; original no. NTR5480; new no. NL5380).

### Funding, and patient and public involvement

This study was performed with funding from ZonMw, the Netherlands Organisation for Health Research and Development (project no. 843002605). The Dutch gynaecological patients’ association agreed upon the design of the study and the grant proposal for funding. No core outcome set has been used in this study.

## Results

**Figure 2:**
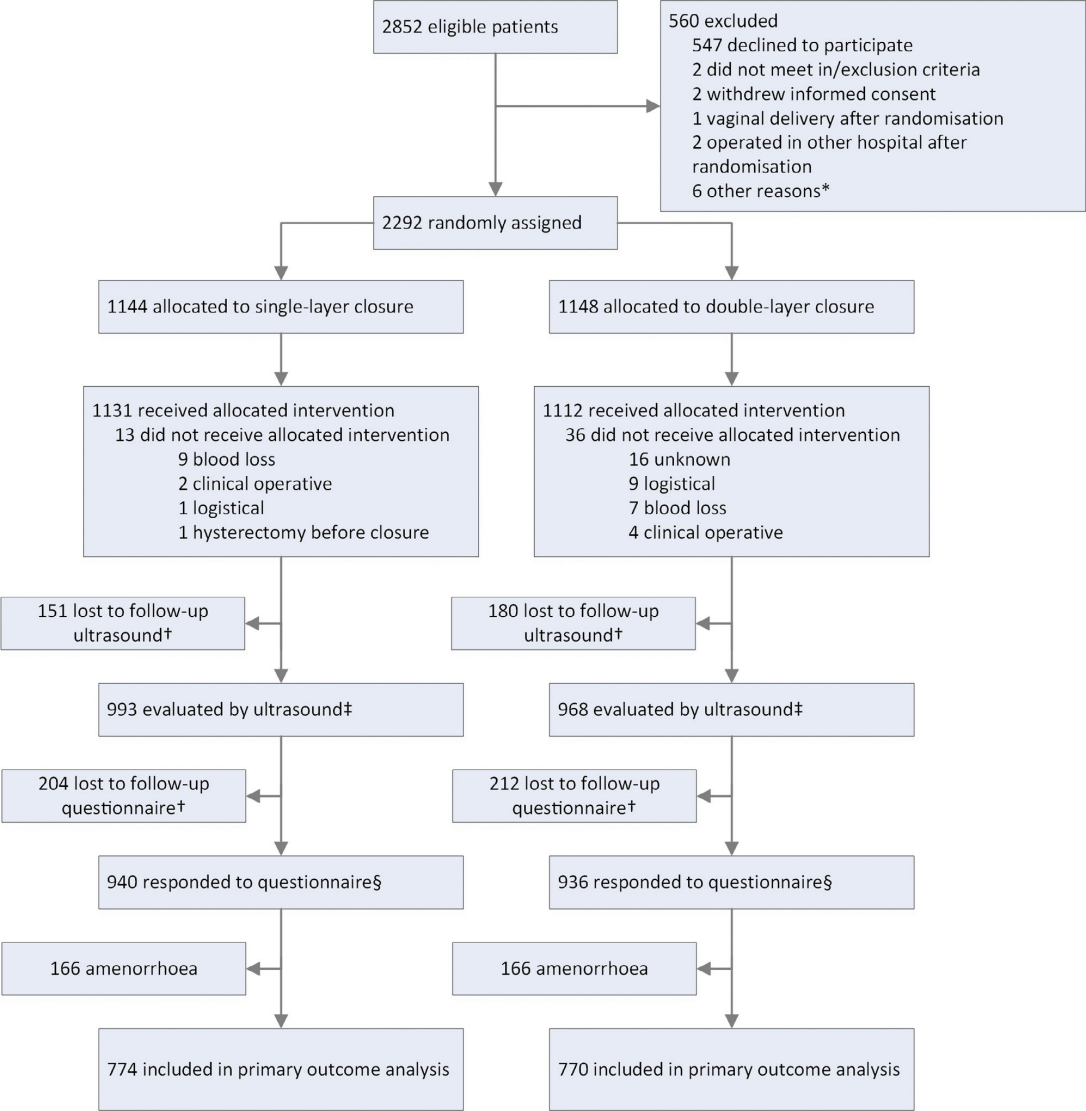
Trial profile. *Logistical reasons, computer randomisation issues, passing through the allocated method to operating gynaecologist or participant not traceable. †Not mutually exclusive: an overlap between groups is possible. ‡Secondary sonographic outcomes reported for this group. §Secondary patient reported outcomes not related to menstruation reported for this group.

Figure 2 shows the trial profile. Between 25 May 2016 and 27 June 2018, 2856 women were found to be eligible (Figure 2). We randomised 2292 (80.4%) of 2852 women across 32 maternity units (Table S2). Thousand one hundered and forty four women were allocated to single‐layer closure and 1148 women were allocated to double‐layer closure of the uterine incision, with 774 and 770 included in the primary outcome analysis, and with 993 and 968 included in analysis of ultrasound outcomes, respectively. Follow‐up ended on 28 May 2019.

Baseline characteristics were similar between the two groups. In total, 1399 (61.0%) participants underwent a prelabour CS for the reasons specified in Table 1: 694 (60.7%) women were in the single‐layer group and 705 (61.4%) women were in the double‐layer group. Perioperative baseline characteristics, including suture material and surgical experience, are presented in Table S3.

**Table 1:** Baseline characteristics

	Variables	Single‐layer closure (*n* = 1144)	Double‐layer closure (*n* = 1148)
**Patient‐reported** *	Age, years	32.0 (4.7)	32.1 (4.6)
	Body‐mass index (kg/m^2^)	26.4 (4.6)	26.6 (4.8)
	**Continent of origin**		
	Europe	963 (95.1%)	948 (93.4%)
	Asia	20 (2.0%)	33 (3.3%)
	Central or South America	15 (1.5%)	15 (1.5%)
	Other	15 (1.5%)	19 (1.9%)
	Current smoker	62 (6.1%)	48 (4.7%)
	**Level of education**		
	Low	68 (6.7%)	70 (6.9%)
	Middle	349 (34.5%)	329 (32.4%)
	High	591 (58.3%)	606 (59.7%)
	Other	5 (0.5%)	10 (1.0%)
	Nulliparous	764 (76.3%)	764 (76.2%)
	Previous miscarriage or abortion	335 (33.1%)	313 (30.8%)
	Underwent curettage, *n*/*N* (%)	165/335 (49.3%)	149/312 (47.8%)
	Previous ectopic pregnancy	13 (1.3%)	18 (1.8%)
	Gestational age at CS (weeks)	39.0 (38.0–39.7)	39.0 (38.0–39.7)
	Preterm delivery < 37 weeks	133 (13.2%)	142 (14.0%)
	Hypertensive disorder**	191 (18.9%)	174 (17.2%)
	Diabetes (mellitus or gestational)	113 (11.2%)	90 (8.9%)
**Characteristics from hospital file**	Twin pregnancy	80 (7.0%)	91 (7.9%)
	**Prelabour caesarean section, reason**	694 (60.7%)	705 (61.4%)
	Breech presentation, *n*/*N* (%)	373/694 (53.7%)	395/705 (56.0%)
	Placenta praevia, *n*/*N* (%)	62/694 (8.9%)	56/705 (7.9%)
	Traumatic vaginal delivery in the past, *n*/*N* (%)	61/694 (8.8%)	60/705 (8.5%)
	Twin pregnancy, *n*/*N* (%)	42/694 (6.1%)	37/705 (5.2%)
	Other, *n*/*N* (%)	156/694 (22.5%)	157/705 (22.3%)
	**Intrapartum caesarean section, reason**	450 (39.3%)	443 (38.7%)
	Failure to progress in 1st stage, *n*/*N* (%)	247/450 (54.9%)	265/443 (59.7%)
	Failure to progress in 2nd stage, *n*/*N* (%)	89/450 (19.8%)	77/443 (17.3%)
	Fetal compromise, *n*/*N* (%)	65/450 (14.4%)	62/443 (14.0%)
	Failed induction, *n*/*N* (%)	18/450 (4.0%)	17/443 (3.8%)
	Other, *n*/*N* (%)	31/450 (7.1%)	23/443 (5.2%)
	Induction of labour	295 (25.8%)	283 (24.7%)
	Received augmentation with oxytocin	377 (33.0%)	393 (34.2%)
	Contractions present	503 (44.0%)	533 (46.4%)
	Dilatation present	479 (41.9%)	494 (43.0%)
	**Dilatation cm**	5 (4–9)	6 (4–9)
	≤3 cm, *n*/*N* (%)	102/478 (21.3%)	78/493 (15.8%)
	4–7 cm, *n*/*N* (%)	212/478 (44.4%)	253/493 (51.3%)
	≥8 cm, *n*/*N* (%)	164/478 (34.3%)	162/493 (32.9%)
	**Fetal station at moment of decision**		
	Elective CS, station unknown, *n*/*N* (%)	594/1070 (55.5%)	571/1087 (52.5%)
	Hodge 0–1, *n*/*N* (%)	339/1070 (31.7%)	377/1087 (34.7%)
	Hodge 2, *n*/*N* (%)	114/1070 (10.7%)	120/1087 (11.0%)
	Hodge 3–4, *n*/*N* (%)	23/1070 (2.1%)	19/1087 (1.7%)
	Emergency CS***	80 (7.0)	90 (7.8)

Data are means (±SDs), *n* (%) or medians (IQRs), unless otherwise indicated; *N* is equal to the total number of patients in the group, unless otherwise indicated; CS, caesarean section.

* Data available for 1013 (88.5%) women from the single‐layer group and 1015 (88.4%) women from the double‐layer group.

** Defined as pregnancy‐induced hypertension or pre‐eclampsia/HELLP (haemolysis, elevated liver enzymes, low platelets) syndrome.

*** Defined as severe fetal distress or maternal disease requiring immediate delivery within several minutes.

The median number of postmenstrual spotting days was 0 (IQR 0–1, range 0–31) in the double‐layer group and 0 (IQR 0–2, range 0–30) in the single‐layer group (adjusted median difference 0.0, 95% CI −0.08 to 0.08, *P* = 1.0; Table 2). An unadjusted analysis for the primary outcome with Mann–Whitney *U*‐test also showed no difference (*P* = 0.643). The mean number of days of postmenstrual spotting was 1.33 (bootstrapped 95% CI 1.12–1.54) for single‐layer closure and 1.26 (bootstrapped 95% CI 1.07–1.45) for double‐layer closure (mean difference −0.07, bootstrapped 95% CI −0.37 to 0.22, *P* = 0.81). As the skewness of the data was greater and the mean number of days with postmenstrual spotting was lower than expected, we performed post‐hoc sensitivity analyses where we dichotomised the number of days with postmenstrual spotting as being present or absent. These analyses also failed to reveal any differences between the groups (Table 2), but did show that spotting was present in approximately 35% of the women. When present, the mean number of days of postmenstrual spotting was 3.75 (SD 4.10) for single‐layer and 3.68 (SD 3.67) for double‐layer closure (*P* = 0.824).

**Table 2:** Menstrual outcomes.

Variables	Single‐layer closure (*n* = 1144)	Double‐layer closure (*n* = 1148)	Outcome measure	Adjusted effect estimate (95% CI)*	*P*
Postmenstrual spotting, days/month	n = 774	n = 770			
Median (IQR)	0.0 (0.0–2.0)	0.0 (0.0–1.0)	Median difference	0.00 (−0.08 to 0.08)	1.0
Mean (SD)	1.33 (3.00)	1.26 (2.77)	Mean difference	−0.07 (−0.37 to 0.22)	0.810
Present at least one day per month	275/774 (35.5%)	264/770 (34.3%)	Relative risk Risk difference	0.96 (0.84 to 1.10) −0.01 (−0.06 to 0.04)	0.580 0.602
Total days blood loss, days/month	6.0 (3.5)	5.9 (3.0)	Mean difference	−0.1 (−0.4 to 0.3)	0.752
Duration of menstruation, days/month	5.5 (2.3)	5.7 (2.5)	Mean difference	0.1 (−0.1 to 0.4)	0.276
Dysmenorrhoea, scale 0–10	4.0 (2.0–6.0)	4.0 (2.0–6.0)	Median difference	0.0 (−0.4 to 0.4)	1.0
Need for treatment of gynaecological complaints**	13/913 (1.4%)	25/903 (2.8%)	Relative risk Risk difference	1.93 (0.99 to 3.75) 0.02 (0.00 to 0.03)	0.052
Lost to follow‐up	204 (17.8%)	212 (18.5%)	Relative risk Risk difference	1.04 (0.88 to 1.24) 0.01 (−0.03 to 0.04)	0.655 0.770
Amenorrhoea	166 (14.5%)	166 (14.5%)	Relative risk Risk difference	1.01 (0.83 to 1.22) 0.00 (−0.03 to 0.03)	0.952 0.997

Data are medians (IQRs), means (SDs) or *n* (%), unless otherwise indicated; 95% CI, 95% confidence interval; CS, caesarean section.

* Adjusted for timing of CS.

** Data available for 913 women in the single‐layer group and 903 women in the double‐layer group; complaints for abdominal pain or abnormal uterine bleeding, treatment could be medicinal, surgical, combined or ‘other’.

Postmenstrual spotting (mean and median) was primary outcome and all others were secondary outcomes. Secondary outcomes. Confidence intervals and *P* values for mean difference in spotting days per month have been determined using bootstrapping. Effect estimates are calculated with single‐layer closure as the reference group.

The proportion of women needing treatment for gynaecological complaints was significantly higher after double‐layer closure (2.8%) than after single‐layer closure (1.4%) when considering the risk difference of 0.02 (95% CI 0.00–0.03, *P* = 0.04), but did not reach significance when considering the relative risk of 1.93 (95% CI 0.99–3.75, *P* = 0.052). Other menstrual characteristics at 9 months after CS did not differ between the groups. We specifically found no difference in dysmenorrhoea score (adjusted median difference 0.0, 95% CI −0.4 to 0.4, *P* = 1.0; Table 2).

The operative time was longer in the double‐layer group (adjusted mean difference 3.9 minutes, 95% CI 3.0–4.9 minutes, *P* < 0.001; Table 3), but no other differences in perioperative outcomes were observed (Table 3).

**Table 3:** Perioperative and sonographic outcomes.

Variables	Single‐layer closure (*n* = 1144)	Double‐layer closure (*n* = 1148)	Outcome measure	Adjusted effect estimate (95% CI)^a^	*P*
**Perioperative outcomes**					
Operative time, minutes from incision to closure	38.9 (11.7)	42.8 (11.2)	Mean difference	3.9 (3.0 to 4.9)	<0.001
Blood loss (mL), geometric mean (95% CI)	405 (392 to 420)	415 (400 to 430)	Geometric mean ratio	1.02 (0.97 to 1.07)	0.384
Need for additional haemostatic sutures	462 (40.5%)	462 (40.4%)	Relative risk Risk difference	1.00 (0.90 to 1.10) −0.00 (−0.04 to 0.04)	0.948 0.981
Number of additional haemostatic sutures needed^b^	1 (1–2)	1 (1–2)	Median difference	0.0 (−0.1 to 0.1)	1.0
Hospital stay, days	3.0 (2.0–4.0)	3.0 (2.0–3.0)	Median difference	0.0 (−0.1 to 0.1)	1.0
Complication rate^c^	118 (10.3%)	104 (9.1%)	Relative risk Risk difference	0.88 (0.69 to 1.13) −0.01 (−0.04 to 0.01)	0.320 0.293
Readmission rate	13/1013 (1.3%)	13/1015 (1.3%)	Relative risk Risk difference	1.00 (0.47 to 2.14) 0.00 (−0.01 to 0.01)	0.996 0.973
**Sonographic outcomes,** ^d^ ***n***	993 (86.8%)	968 (84.3%)	—	—	—
Niche prevalence	684 (68.9%)	712 (73.6%)	Relative risk Risk difference	1.06 (1.01 to 1.13) 0.05 (0.01 to 0.09)	0.033 0.027
RMT, mm	6.4 (3.3)	6.7 (3.4)	Mean difference	0.3 (−0.1 to 0.6)	0.108
RMT/AMT ratio	0.56 (0.41–0.75)	0.58 (0.41–0.74)	Median difference	0.01 (−0.02 to 0.04)	0.550
Large niche prevalence (RMT ≤ 3 mm)	131 (13.2%)	113 (11.8%)	Relative risk Risk difference	0.89 (0.70 to 1.12) −0.01 (−0.04 to 0.02)	0.310 0.657
Large niche prevalence (RMT/AMT ratio < 50%)	351 (35.6%)	337 (35.1%)	Relative risk Risk difference	0.98 (0.87 to 1.11) −0.00 (−0.05 to 0.04)	0.792 0.870
Niche depth (mm), geometric mean (95% CI)^e^	3.93 (3.81 to 4.06)	3.95 (3.83 to 4.07)	Geometric mean ratio	1.02 (0.97 to 1.08)	0.498
Niche length (mm), geometric mean (95% CI)^e^	4.72 (4.53 to 4.91)	4.95 (4.78 to 5.13)	Geometric mean ratio	1.05 (0.99 to 1.11)	0.056
Niche width (mm), geometric mean (95% CI)^e^	5.04 (4.83 to 5.26)	5.08 (4.87 to 5.29)	Geometric mean ratio	1.01 (0.95 to 1.07)	0.737
Niche volume^f^ (mm^3^), geometric mean (95% CI)^e^	22.9 (20.9 to 25.2)	25.4 (23.2 to 27.7)	Geometric mean ratio	1.11 (0.98 to 1.27)	0.106
Presence of intracavitary fluid^g^	141 (14.2%)	157 (16.3%)	Relative risk Risk difference	1.14 (0.92 to 1.40) 0.02 (−0.01 to 0.05)	0.222 0.256
Uterine position					
Anteverted uterine position	696 (70.4%)	688 (71.7%)	Reference category	—	—
Stretched uterine position	65 (6.6%)	63 (6.6%)	Relative risk Risk difference	1.00 (0.72 to 1.40) −0.0 (−0.02 to 0.02)	0.993 0.910
Retroverted uterine position	193 (19.5%)	178 (18.5%)	Relative risk Risk difference	0.95 (0.79 to 1.14) −0.01 (−0.05 to 0.03)	0.582 0.581
Extremely retroverted (angle < 45°)	34 (3.4%)	31 (3.2%)	Relative risk Risk difference	0.95 (0.59 to 1.53) −0.00 (−0.15 to 0.15)	0.822 0.994

Data are means (SDs), *n* (%) or medians (IQRs), unless otherwise indicated; *N* represents the number of women with data available; 95% CI, 95% confidence interval; CS, caesarean section; AMT, adjacent myometrial thickness; RMT, residual myometrium thickness.

^a^ Adjusted for timing of CS.

^b^ Only recorded when at least one additional haemostatic suture was needed.

^c^ Fever, bladder/intestinal lesion, postpartum haemorrhage or other.

^d^ Ultrasound outcomes measured at 3 months of follow‐up and presented as smallest RMT or largest niche measurement, either from transvaginal ultrasound or contrast‐enhanced ultrasound, when available.

^e^ Only recorded when a niche was present.

^f^ Calculated as 1/3 × π × (1/2 × length)^2^ × depth.

^g^ Only available from transvaginal ultrasound, not when contrast was used. Effect estimates are calculated with single‐layer closure as the reference group.

We found a statistically significant difference in niche prevalence at TVUS evaluation at three months postoperatively: 684 (68.9%) of 993 women in the single‐layer group versus 712 (73.6%) of 968 women in the double‐layer group had a niche of ≥ 2 mm in depth (adjusted RR 1.06, 95% CI 1.01–1.13, *P* = 0.033; Table 3). No significant differences were found in the prevalence of large niches, in residual myometrium thickness (RMT) or in the other sonographic secondary outcomes (Table 3).

The change in general health, a subdomain of health‐related quality of life, from 3 to 9 months of follow‐up was worse after double‐layer closure: median change score 0 (IQR −10 to 5) after single‐layer closure and −5 (IQR −15 to 5) after double‐layer closure (*P* = 0.03). The change in social functioning was also worse after double‐layer closure, reflected in the interquartile range: median change score 0 (IQR 0–12.5) after single‐layer closure and 0 (IQR −12.5 to 12.5) after double‐layer closure (*P* = 0.016) (Table S4). Sexual function at 9 months of follow‐up was rated worse after double‐layer closure on the subdomains of satisfaction (adjusted median difference −0.4, IQR −0.6 to −0.2, *P* = 0.001) and pain (adjusted median difference −0.4, IQR −0.8 to 0.0, *P* = 0.03) (Table S3). No other differences were found in health‐related quality of life, social participation or sexual functioning (Tables S4–S6).

Thirty‐seven (1.6%) serious adverse events were reported (*n* = 23 in the single‐layer group and *n* = 14 in the double‐layer group), including hospital admissions or prolonged hospitalisation as a result of infection, ileus, postpartum haemorrhage and recovery from pre‐eclampsia or HELLP syndrome (haemolysis, elevated liver enzymes and low platelets). All but one of the serious adverse events were judged as complications related to the CS in general and were equally distributed among groups.

Per‐protocol analyses revealed that the proportion needing treatment for gynaecological complaints also differed when considering the relative risk (1.4% after single‐layer closure and 2.9% after double‐layer closure, RR 1.97, 95% CI 1.01–2.82, *P* = 0.045; Table S9). No other differences were revealed compared with ITT analyses (Tables S9 and S10).

Subgroup analyses did not reveal any difference in the effect of closure technique on primary outcome between prespecified subgroups (Table S7).

Within the single‐layer group, the endometrial handling technique (i.e. including or excluding the endometrium in the suture) was recorded for 1036 women (90.6%): 743 (71.7%) women received uterine closure including the endometrium; and 293 (28.3%) women received uterine closure excluding the endometrium. The median number of days of postmenstrual spotting did not differ: 0 (IQR 0–1.5) in the group including the endometrium and 0 (IQR 0–2) in the group excluding the endometrium (adjusted median difference 0.0, 95% CI −0.3 to 0.3, *P* = 1.0; Table S8). Niche prevalence was lower after single‐layer uterine closure excluding the endometrium (150/293, 59.3%) compared with single‐layer uterine closure including the endometrium (471/743, 71.8%) (adjusted RR 0.83, 95% CI 0.74–0.93, *P* = 0.001; Table S8). Dysmenorrhoea, the prevalence of large niches and RMT/adjacent myometrium thickness (AMT) ratio was equal for both subgroups within the single‐layer closure arm.

In the double‐layer group, the total societal costs were on average €5711 (standard error, SE €304), versus €5408 (SE €276) in the single‐layer group, with no significant difference (mean difference €303, 95% CI −€413 to €1035). There was also no significant difference in the number of quality‐adjusted life years (QALYs) gained between the arms: 0.659 QALYs (SE 0.003 QALYs) for the double‐layer group and 0.663 QALYs (SE 0.003 QALYs) for the single‐layer group, with a mean difference of −0.004 QALYs (95% CI −0.013 to 0.005 QALYs). Further details will be published in a separate article.

## Discussion

### Main findings

In this large double‐blind randomised multicentre trial, we found no difference in the number of days of postmenstrual spotting during a single menstrual cycle 9 months after a first CS between women randomised to a single‐layer unlocked uterine closure technique and women randomised to a double‐layer unlocked uterine closure technique. The single‐layer technique was found to have some small positive effects on a selection of secondary outcomes, including shorter operative time, lower niche prevalence, less need for treatment of gynaecological complaints, less reported intercourse‐related pain and higher sexual satisfaction, and less deterioration in general health and social functioning. Furthermore, double‐layer closure was not cost‐effective in terms of societal costs or QALYs gained.

### Strengths and limitations

The major strengths of our study are the large sample size, the execution of the trial according to a previously published protocol, the mandatory trial‐specific instruction video for performing the intervention and the high response rate for our digital questionnaire and TVUS follow‐up. We excluded women with a previous CS and we used no locked sutures, as both factors are associated with higher niche prevalence and therefore possibly with clinical outcomes. Additionally, sonographers were required to complete a digital e‐learning module on niche measurements, based on the consensus of a European expert panel.^7^ A final strength is our primary outcome, which is a clinical outcome measure reported by patients, instead of using a possible sonographic surrogate for gynaecological symptoms or future obstetric complications. The proportion of women unevaluable for the primary outcome, mainly as a result of amenorrhoea, was estimated beforehand (i.e. 35%).

Our study also has some limitations. First, not all gynaecologists always adhered to the allocated technique (*n* = 13, 1.1%, in the single‐layer group; *n* = 36, 3.1%, in the double‐layer group), although per‐protocol analyses revealed no large differences compared with ITT analyses. Second, the sonographic evaluation was performed at 3 months of follow‐up, which is possibly early in the healing process, as previous studies have shown that the appearance of the scar changes – and specifically RMT decreases – over time.^21^ Experience in performing TVUS could have played a role, but a post‐hoc sensitivity analysis for the secondary outcome of niche prevalence yielded a similar RR for the effect of closure technique used, after additional correction for the hospital being an expertise centre in niche diagnostics or not. Another limitation is that sonohysterography was only used when a niche was not visible with TVUS, in order to reduce costs and patient discomfort, but from a research perspective using sonohysterography for all women might have been preferable.^20^ Our study was by definition limited through dichotomising the two surgical techniques into a simple ‘single’ and ‘double’ layer. It is likely that other and more difficult to measure constituents of the techniques play an important role in wound healing and the formation of a niche. Lastly, the single‐layer closure is the standard uterine closure technique in the Netherlands, which could have influenced the results.

### Interpretation (in light of other evidence)

The findings of our trial are not in line with our hypothesis that the number of days of postmenstrual spotting would be lower after a double‐layer closure. We based our hypothesis on previous studies showing that large niches are related to a higher prevalence of postmenstrual spotting,^4^, ^5^ and on our meta‐analysis in which sonographic findings, suggested to be intermediates for clinical symptoms, were better after double‐layer closure.^15^ We have confirmed, however, that the similar percentage of large niches and thickness of residual myometrium in our population corresponded with an equal number of days of postmenstrual spotting for both groups.

Studies comparing uterine closure techniques often lack an evaluation of gynaecological symptoms. A recently published small randomised controlled trial (RCT, *n* = 138) compared a single‐layer locked closure technique with a double‐layer unlocked closure technique, and reported a significantly lower ‘prevalence of postmenstrual spotting’ after the double‐layer closure, but a precise definition was lacking.^22^ In a previous pooled analysis dysmenorrhoea was more prevalent after a single‐layer closure (RR 1.23, 95% CI 1.01–1.48, *n* = 7484) compared with a double‐layer closure,^3^, ^23^ but these two studies were heterogeneous in terms of the moment at which dysmenorrhoea was reported.^15^ The present trial shows no differences in median postmenstrual spotting days or dysmenorrhoea scores between the two arms.

The current results are in line with our previously published systematic review concerning short‐term perioperative outcomes, dominated by the largest trial on this topic (CORONIS): slightly longer operative time for double‐layer closure and no differences in maternal complications.^15^, ^24^

Ultrasound evaluation showed that the prevalence of large niches and RMT did not differ between the arms of our trial, which is in line with previous studies.^15^, ^23^, ^25^ The similar percentage of large niches was reflected in the similar number of days, and equal percentage presence, of postmenstrual spotting. On the other hand, more niches were present with a cut‐off value of 2 mm after double‐layer closure, whereas previous studies found no difference in niche prevalence.^15^, ^16^, ^23^ A possible explanation is that no clear definition or cut‐off for niches was used in previous studies, or that our sample size is considerably larger than previously executed studies (*n* = 61 and *n* = 267).^16^, ^23^ The default inclusion of endometrium in our double‐layer group, or introducing more suture material, causing greater uterine tissue disruption, could have led to less optimal wound healing and increased niche prevalence.^25^ The size of a niche and the thickness of the residual myometrium are considered to be more important surrogate markers for detrimental effects later in life, however, rather than the presence of a smaller niche.^4^, ^5^, ^6^, ^9^, ^10^ Other secondary outcomes (health‐related quality of life, sexual functioning, social participation) have not been reported previously in this context, and nor has cost‐effectiveness.

Another contradictory ultrasound finding is a higher niche prevalence when the endometrium was included in the single‐layer group, whereas a higher niche prevalence when excluding the endometrium was reported by Yazicioglu et al.^26^ They randomised patients to inclusion or exclusion of the endometrium, whereas we let gynaecologists choose the preferred technique, possibly introducing selection bias. Advanced stage of labour as a patient‐related factor or the accurate approximation of the uterine layers as a surgeon‐related factor have not been taken into account in our comparison.

The results of our study are generalisable to the general pregnant European population who will undergo a first CS, either as an elective procedure or during labour. Caution should be taken to extrapolate our results to a non‐European population, or to women who undergo repeat CSs or who otherwise did not fulfil our criteria (e.g. having previous uterine surgery or menstrual disorders with known cause).^12^ Furthermore, through an overrepresentation of planned CS in the responder group (see Table S11), our results show an underestimation of niche‐related postmenstrual spotting, which decreases the generalisability (for more information, see Appendix S2).

Regarding future research, we will first of all wait for the results of our 3‐year follow‐up to report on important gynaecological, fertility and obstetric outcomes. Furthermore, the identification of risk factors and thereby other strategies to reduce niche prevalence should be a focus of future research, as a niche in the caesarean scar is an underestimated contributor to long‐term maternal morbidity. Postulated protective factors are higher level of hysterotomy and the use of adhesion barriers.^14^, ^27^ An unclear factor is the inclusion or exclusion of the endometrium. All of these factors should be investigated further in future large RCTs with clinical maternal symptoms as the primary outcome.

## Conclusion

The results of our trial show no superiority of double‐layer uterine closure after a first CS in terms of postmenstrual spotting at 9 months of follow‐up. In contrast, double‐layer closure appears slightly unfavourable in terms of operative time, niche prevalence, required treatment of gynaecological symptoms, sexual functioning, social functioning and general health. In terms of postmenstrual spotting, there is no evidence to support double‐layer closure in a comparable population. As the prevalence of large niches and RMT did not differ, we do not expect differences in long‐term fertility and obstetric outcomes between the groups, but we cannot state this with certainty until the long‐term results of our trial are available. If no superiority of double‐layer closure is demonstrated over the long‐term follow‐up, and specifically for risk of uterine rupture, the guidelines should be adjusted accordingly.

### Disclosure of interests

JAFH received grants from ZonMw, during the conduct of the study, grants from Samsung and PlantTec Medical GmbH, and a fee from Olympus, outside the submitted work. MYB received a grant from the Leading the Change Project, outside the submitted work. CBL received departmental grants from Ferring, Merck and Guerbet, all outside the submitted work. CJMdG received a grant from ZonMw outside the submitted work. All other authors declare no competing interests. Completed disclosure of interests forms are available to view online as supporting information.

### Contribution to authorship

SIS collected data, analysed and interpreted data, and drafted the report. LFvdV designed the study, collected data, and participated in drafting and revising the report. RAdL created multiple tools essential for trial execution and participated in drafting and revising the report. AJB, PMvdV and RGD analysed and interpreted the data, and participated in drafting and revising the report. MYB, CBL and CJMdG designed the study and critically revised the first draft of the report. JAFH designed the study, was principle investigator, interpreted the data, and participated in drafting and revising the report. All authors approved the final version of the article for publication.

### Details of ethics approval

The study was approved by the Institutional Review Board (IRB) of Amsterdam UMC, location VU University Medical Centre, in December 2015 (registration no. 2015.462), and by the boards of all participating hospitals before the start of inclusion. No substantial changes were made to the protocol after commencement of the trial. All participants provided written informed consent before taking part in the study.

### Funding

This study was performed with funding from ZonMw: The Netherlands Organisation for Health Research and Development (project no. 843002605). Both the funder of the study and a patient involvement panel approved the study protocol. The funder had no role in study design, data collection, data analysis, data interpretation or writing the article. The corresponding author had full access to all of the data in the study and had final responsibility for the decision to submit for publication.

### Acknowledgements

We would like to thank all participants of the 2Close study. Additionally, we would like to thank the Departments of Obstetrics and Gynaecology of all participating hospitals, with special thanks to all research nurses and research midwives for their contribution in data collection. The 2Close study group would like to thank all collaborators from the participating hospitals for their effort.

## Supporting information


**Figure S1.** Double‐layer uterine closure with a standardised method.
**Figure S2.** Examples of e‐learning niche measurement.

**Table S1.** Secondary outcome measures.
**Table S2.** Number of participants enrolled by hospital.
**Table S3.** Perioperative baseline characteristics.
**Table S4.** 36‐Item Short Form Health Survey (SF‐36).
**Table S5.** PROMIS Ability to Participate in Social Roles and Activities v2.0 short form 8a (PROMIS‐APS).
**Table S6**. Female Sexual Function Index (FSFI).
**Table S7.** Subgroup analyses.
**Table S8.** Outcomes within single‐layer group, with or without application of endometrial saving technique.
**Table S9.** Per‐protocol analyses for all outcomes (except SF‐36).
**Table S10.** Per‐protocol analyses for SF‐36.
**Table S11.** Differences in baseline characteristics between responders and non‐responders for the primary outcome.

**Appendix S1.** Further discussion.

Supplementary Material

Supplementary Material

Supplementary Material

Supplementary Material

Supplementary Material

Supplementary Material

Supplementary Material

Supplementary Material

Supplementary Material

Supplementary Material

## Data Availability

De‐identified individual participant data collected during the 2Close trial will be shared 1 year after publication of the long‐term results on request (j.huirne@amsterdamumc.nl). Approval of a proposal will be necessary before data will be shared. To gain access, requesters will need to sign an agreement form and confirm that the data will be used for the purpose for which access was granted.

## The 2Close study group

**Table:**

Site principal investigator	Hospital
Marchien van Baal	Flevo ziekenhuis, Almere
Erik van Beek	Sint Antonius ziekenhuis, Nieuwegein
Mireille N Bekker	Birth Centre Wilhelmina Children Hospital/University Medical Centre Utrecht, Utrecht
Karin de Boer	Rijnstate hospital, Arnhem
Elisabeth MA Boormans	Meander Medical Centre, Amersfoort
Judith E Bosmans	VU University, dept. of Health Sciences
Hugo WF van Eijndhoven	Isala clinics, Zwolle
Mohamed El Alili	VU University, dept. of Health Sciences
Hanneke Feitsma	Haga hospital, Den Haag
Wouter JK Hehenkamp	Amsterdam UMC, VU University, Amsterdam
Majoie Hemelaar	Dijklander hospital – location Hoorn
Wietske Hermes	Haaglanden Medical Centre – Westeinde hospital, Den Haag
Esther Hink	Radboud University Nijmegen Medical Centre, Nijmegen
Anjoke JM Huisjes	Gelre hospital – location Apeldoorn
Ineke Janssen	Groene Hart hospital, Gouda
Kitty Kapiteijn	Reinier de Graaf hospital, Delft
Mesrure Kaplan	Röpcke‐Zweers hospital, Hardenberg
Paul JM van Kesteren	OLVG‐oost, Amsterdam
Judith OEH van Laar	Máxima Medical Centre, Veldhoven
Josje Langenveld	Zuyderland Medical Centre, Heerlen
Wouter J Meijer	Gelre hospital – location Zutphen
Angèle LM Oei	Bernhoven hospital, Uden
Eva Pajkrt	Amsterdam UMC, Univ of Amsterdam, Amsterdam
Dimitri NM Papatsonis	Amphia hospital, Breda
Celine M Radder	OLVG‐west, Amsterdam
Robbert JP Rijnders	Jeroen Bosch hospital, 's‐Hertogenbosch
Liesbeth Scheepers	Maastricht University Medical Centre, Research school ‘GROW’, Maastricht
Daniela H Schippers	Canisius‐Wilhelmina hospital, Nijmegen
Nico WE Schuitemaker	Diakonessenhuis, Utrecht
Marieke Sueters	Leiden University Medical Centre, Leiden
Harry Visser	Tergooi hospital, Blaricum
Huib AAM van Vliet	Catharina hospital, Eindhoven
Marloes de Vleeschouwer	Sint Franciscus Hospital, Rotterdam
