# Supplementary material for: Effect of single‐ versus double‐layer uterine closure during caesarean section on postmenstrual spotting (2Close): multicentre, double‐blind, randomised controlled superiority trial
Source: BJOG. 2020 Oct 25;128(5):866–78. doi: 10.1111/1471-0528.16472 (PMC7983985; doi:10.1111/1471-0528.16472)
Supplement: Supplementary file 1 — Figure S1. Double‐layer uterine closure with a standardised method. Figure S2. Examples of e‐learning niche measurement. [file BJO-128-866-s003.pdf]

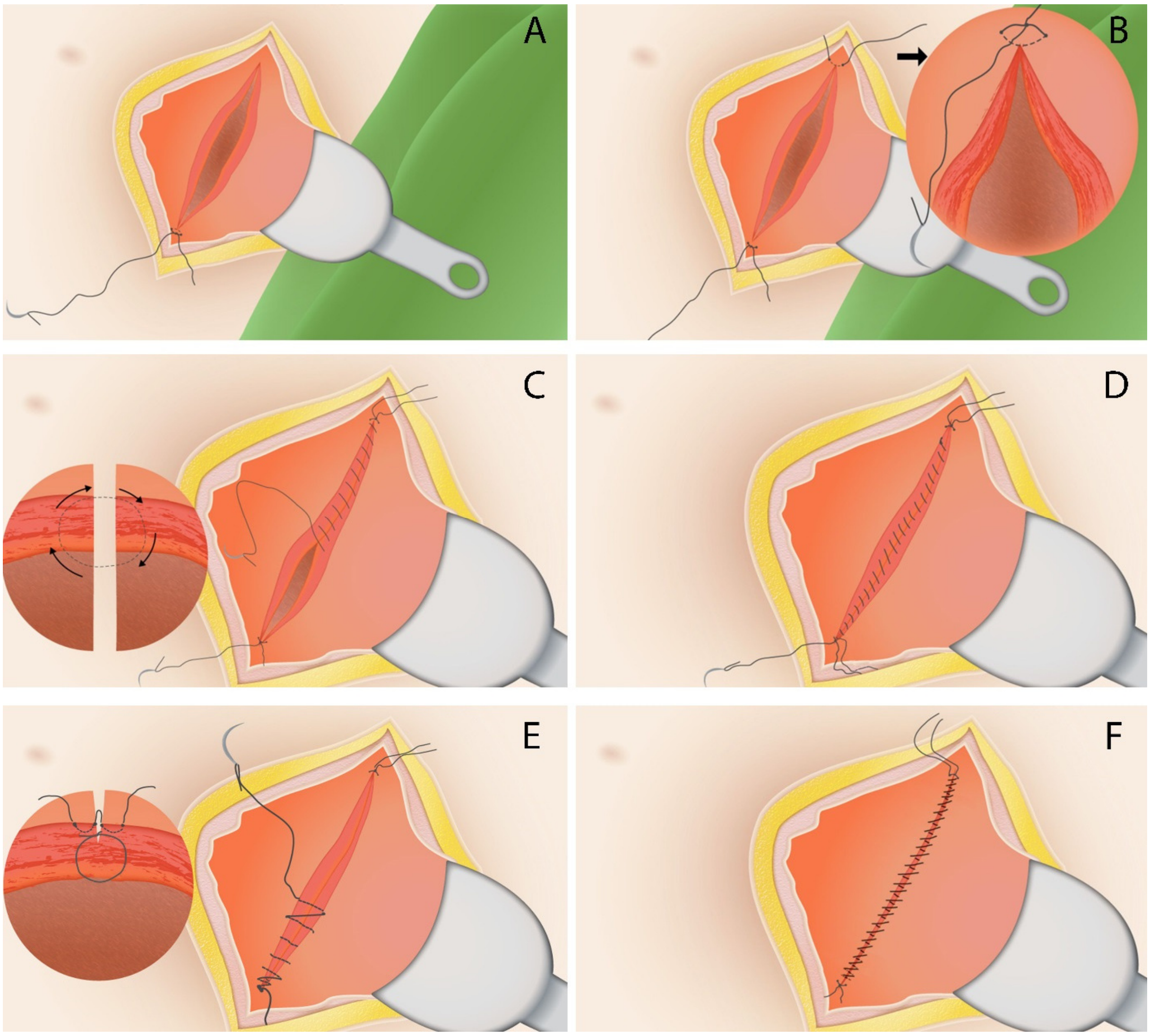

Figure S1. Double-layer uterine closure in a standardised way.

A. Step 1: lateral suture; B. Step 2: lateral suture on the other side; C. Step 3: First layer: full thickness, continuous, including large part of myometrium, including the endometrial layer; D. Step 4: End of this first layer; E. Step 5: Second layer: superficial continuous layer of serosal tissue, imbricating the first layer; F. Step 6: First and second layer should be closely connected.

3. Recognizing a niche – quiz3

Please select the niche

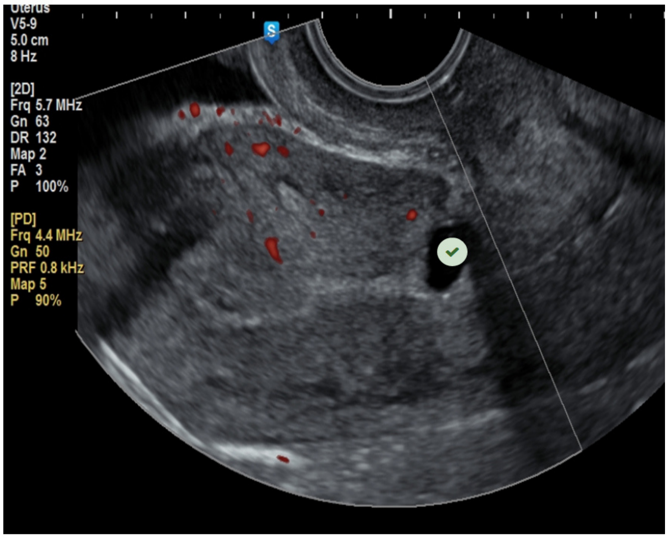

Well done

Lesson 5 – How to measure

1. Lesson 5 – How to measure

1.5. Simple niche – depth

Depth of the niche is measured in one straight line from base shaped niche, the largest depth should be measured.

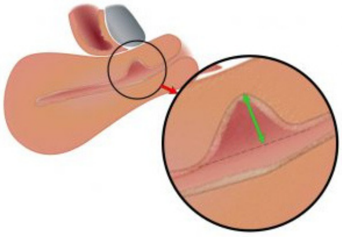

Lesson 4 – Finding the planes

‹ Next→

1. Sagittal plane

The video below shows how to get to the correct sagittal plane for niche measurement. Start with good visualization of the cervical canal, then move the vaginal probe to both lateral sides, as is shown in the video below. Do not rotate the probe.

For depth and length measurement, find the image where the main niche has the largest length and depth. All measurements will be explained later.

Keep in mind that the correct sagittal plane, is the plane with the largest niche. This is NOT always the mid sagittal plane.

Please click on the texts in the video for more details:

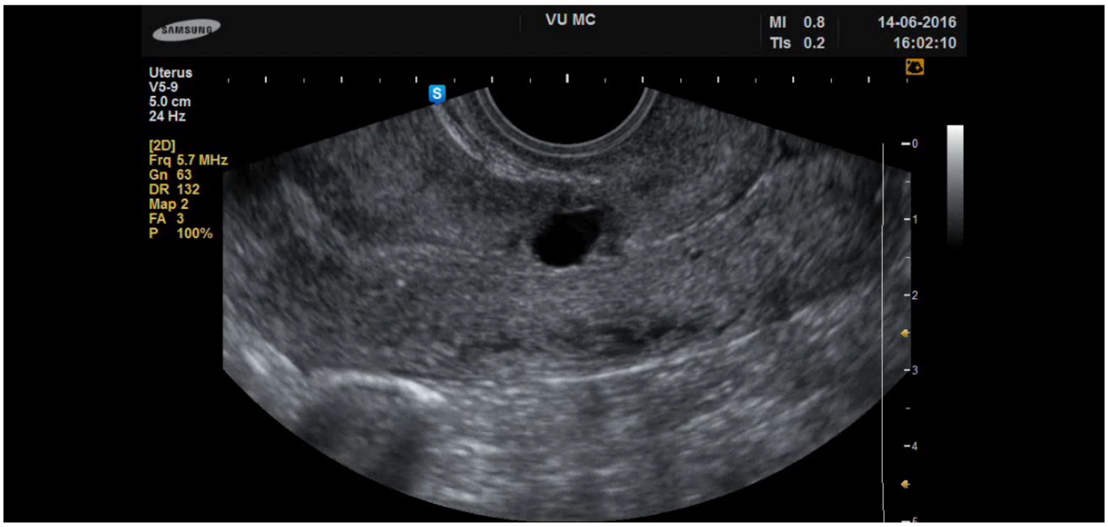

Figure S2. Examples of e-learning niche measurement.
