# Supplementary material for: Effect of single‐ versus double‐layer uterine closure during caesarean section on postmenstrual spotting (2Close): multicentre, double‐blind, randomised controlled superiority trial
Source: BJOG. 2020 Oct 25;128(5):866–78. doi: 10.1111/1471-0528.16472 (PMC7983985; doi:10.1111/1471-0528.16472)
Supplement: Supplementary file 2 — Table S1. Secondary outcome measures. Table S2. Number of participants enrolled by hospital. Table S3. Perioperative baseline characteristics. Table S4. 36‐Item Short Form Health Survey (SF‐36). Table S5. PROMIS Ability to Participate in Social Roles and Activities v2.0 short form 8a (PROMIS‐APS). Table S6. Female Sexual Function Index (FSFI). Table S7. Subgroup analyses. Table S8. Outcomes within single‐layer group, with or without application of endometrial saving technique. Table S9. Per‐protocol analyses for all outcomes (except SF‐36). Table S10. Per‐protocol analyses for SF‐36. Table S11. Differences in baseline characteristics between responders and non‐responders for the primary outcome. [file BJO-128-866-s012.pdf]

Table S1. Secondary outcome measures, measurement instrument and time point of measurement

Outcome	Measurement instrument	Time point
Perioperative data		
Operative time	Operative report	Baseline
Blood loss	Operative report	Baseline
Need for additional hemostatic sutures, including number	Operative report	Baseline
Complications up to 6 weeks	Hospital file	Baseline
Duration of hospital stay	Questionnaire, patient-reported	Baseline
Readmission rate	Questionnaire, patient-reported	Baseline
Menstrual characteristics		
Duration of menstruation	Questionnaire, patient-reported	9 months
Total days of blood loss per month	Questionnaire, patient-reported	9 months
Dysmenorrhoea (scale 0-10)	Questionnaire, patient-reported	9 months
Treatment needed for abdominal pain	Questionnaire, patient-reported	9 months
Treatment needed for bleeding disorder	Questionnaire, patient-reported	9 months
Health related quality of life	SF-36[1]	3 months, 9 months
Self-rated health	EQ-5D-5L[2]	Baseline, 3 months, 9 months
Sexual functioning	FSFI[3]	9 months
Social participation	PROMIS-APS[4]	3 months
Sonographic outcomes	First step: transvaginal ultrasound (TVUS)<br><br>Second step: sonohysterography with either saline or gel	
Presence of a niche	TVUS, contrast enhanced if necessary	3 months
Residual myometrium thickness	TVUS, contrast enhanced if necessary	3 months
Adjacent myometrium thickness	TVUS, contrast enhanced if necessary	3 months
Ratio RMT/AMT	TVUS, contrast enhanced if necessary	3 months
Large niche, defined as RMT <3mm or ratio <50%	TVUS, contrast enhanced if necessary	3 months
Niche depth	TVUS, contrast enhanced if necessary	3 months
Niche length	TVUS, contrast enhanced if necessary	3 months
Niche width	TVUS, contrast enhanced if necessary	3 months
Niche volume, 2 dimensional measurements	$1/3 * \pi * (1/2 * \text{niche length})^2 * \text{niche depth}$	3 months
Presence of intracavitary fluid	TVUS, contrast enhanced if necessary	3 months
Uterine position	TVUS, contrast enhanced if necessary	3 months

Table S2. Number of participants enrolled per site

Hospital	Number of participants enrolled
Amphia hospital, Breda	68
Amsterdam UMC, Univ of Amsterdam, Amsterdam	41
Amsterdam UMC, VU University, Amsterdam	147
Bernhoven hospital, Uden	82
Birth Centre Wilhelmina Children Hospital/University Medical Centre Utrecht, Utrecht	70
Canisius-Wilhelmina hospital, Nijmegen	17
Catharina hospital, Eindhoven	111
Deventer hospital, Deventer	146
Diakonessenhuis, Utrecht	21
Dijklander hospital – location Hoorn	42
Flevo hospital, Almere	141
Gelre hospital – location Apeldoorn	27
Gelre hospital – location Zutphen	14
Groene Hart hospital, Gouda	51
Haaglanden Medical Centre – Westeinde hospital, Den Haag	28
Haga hospital, Den Haag	58
Isala clinics, Zwolle	57
Jeroen Bosch hospital, 's-Hertogenbosch	114
Leiden University Medical Centre, Leiden	62
Maastricht University Medical Centre, Research school 'GROW', Maastricht	56
Máxima Medical Centre, Veldhoven	180
Meander Medical Centre, Amersfoort	89
OLVG-oost, Amsterdam	50
OLVG-west, Amsterdam	70
Radboud University Nijmegen Medical Centre, Nijmegen	54
Reinier de Graaf hospital, Delft	187
Rijnstate hospital, Arnhem	24
Röpcke-Zweers hospital, Hardenberg	23
Sint Antonius Hospital, Nieuwegein	28
Sint Franciscus Hospital, Rotterdam	66
Tergooi hospital, Blaricum	65
Zuyderland Medical Centre, Heerlen	103
Total	2292

Table S3. Perioperative characteristics

	**Single-layer (*n*=1144)**	**Double-layer (*n*=1148)**
**Closure according to randomisation**	1131 (98.9%)	1112 (96.9%)
**Caesarean section performed by**		
Gynaecologist	643 (56.2%)	631 (55.0%)
Resident under supervision	501 (43.8%)	517 (45.0%)
**Antibiotics administrated**	1139 (99.7%)	1141 (99.5%)
**Uterine incision**		
In lower uterine segment	1128 (98.6%)	1135 (99.0%)
Other*	16 (1.4%)	12 (1.0%)
**Bladder flap created**	971 (88.5%)	975 (88.5%)
Missing, n	47	46
**Suture material used**		
Vicryl	903 (79.0%)	912 (79.4%)
Novosyn	217 (19.0%)	214 (18.6%)
PDS	3 (0.3%)	3 (0.3%)
Other	20 (1.7%)	19 (1.7%)
**Endometrial saving technique applied**	293 (28.3%)	NA
**Additional operation in same session**	39 (3.4%)	23 (2.0%)
**Birth weight**		
Singleton pregnancies	3317 (698)	3300 (739)
Twin pregnancies	2402 (598)	2386 (568)

Data are number (%), or mean (SD), unless otherwise indicated. NA=not applicable since double-layer closure was default including endometrium. *Other reasons included low vertical, classical incision, and J-incision.

Table S4. Health-related quality of life scores for single- and double-layer group, and change in score over time

SF-36 domain	Measurement moment	Single-layer (n=1144)*	Double-layer (n=1148)*	p-value for comparison of change between arms
	3 months	52.4 (7.6)	52.1 (7.8)	—
PCS	9 months	54.3 (7.2)	53.6 (7.9)	—
	Change	1.9 (6.0)	1.5 (6.6)	0.366†
	3 months	50.1 (9.9)	50.2 (9.1)	—
MCS	9 months	49.4 (9.4)	48.6 (10.1)	—
	Change	-1.1 (8.7)	-1.6 (8.8)	0.205†
	3 months	95 (85-100)	95 (85-100)	—
Physical functioning	9 months	100 (90-100)	95 (90-100)	—
	Change	5 (0-10)	0 (0-10)	0.398‡
	3 months	100 (50-100)	100 (50-100)	—
Physical role	9 months	100 (100-100)	100 (75-100)	—
	Change	0 (0-25)	0 (0-25)	0.142‡
	3 months	89.8 (67.4-100)	89.8 (67.4-100)	—
Bodily pain	9 months	89.8 (77.6-100)	89.8 (69.4-100)	—
	Change	0 (0-12.2)	0 (-2 - 12.2)	0.731‡
	3 months	75 (65-90)	75 (65-90)	—
General health	9 months	75 (60-85)	70 (55-85)	—
	Change	0 (-10 - 5)	-5 (-15 - 5)	0.030‡
	3 months	100 (66.7-100)	100 (66.7-100)	—
Emotional role	9 months	100 (100-100)	100 (100-100)	—
	Change	0 (0-0)	0 (0-0)	0.161‡
	3 months	60 (45-75)	60 (48.8-70)	—
Energy/vitality	9 months	60 (45-70)	60 (45-70)	—
	Change	0 (-10 - 10)	0 (-10 - 10)	0.697‡
	3 months	84 (72-92)	84 (72-92)	—
Mental health	9 months	80 (72-88)	80 (68-88)	—
	Change	0 (-8 - 4)	0 (-8 - 4)	0.932‡
	3 months	87.5 (62.5-100)	87.5 (62.5-100)	—
Social functioning	9 months	87.5 (75-100)	87.5 (75-100)	—
	Change	0 (0-12.5)	0 (-12.5 - 12.5)	0.016‡

Scores reported as mean (SD), or median (IQR). The Medical Outcomes Study 36-Item Short Form Health Survey (SF-36) general health-related quality of life scores range from 0 (severely affected) to 100 (not affected). CI=confidence interval, PCS=physical component summary score, MCS=mental component summary score. Change=9 months score minus 3 months score. Both summary scores are z-scores compared with Dutch female reference population with mean of 50, standard deviation (SD) of 10. *3 months score available for 972 and 970 women, respectively; 9 months score available for 883 and 872 women, respectively; Change score available for 840 and 832 women, respectively. †p-value for interaction term of treatment arm and measurement moment in linear mixed model; ‡Mann-Whitney test on change scores.

Table S5. Social participation for single-layer and double-layer group

	Single-layer (*n*=1144)*	Double-layer (*n*=1148)*	Adjusted median difference (95% CI)†	p-value
**PROMIS-APS‡**	52.7 (48.0-60.2)	52.7 (47-60.2)	0.0 (-1.0 to 1.0)	1.0

Data are median (IQR). PROMIS-APS=PROMIS Ability to Participate in Social Roles and Activities v2.0 short form 8a. *Data available for 967 and 969 women, respectively. †Adjusted for moment of CS. ‡Scores from PROMIS-APS are expressed as a T-score that represents a standardised score with a mean of 50 (corresponding to the mean score of the general population of the United States) and standard deviation (SD) of 10. Higher scores indicate better ability to participate. Effect estimates are calculated with single-layer as the reference group.

Table S6. Sexual function scores for single-layer and double-layer group, for each domain and full scale score

FSFI domain*	Single-layer (*n*=1144)†	Double-layer (*n*=1148)†	Adjusted median difference (95% CI)‡	p-value
Full scale§	23.2 (19.5-25.9)	22.9 (18.9-25.4)	-0.2 (-0.8 to 0.4)	0.519
Desire	4.2 (3.6-4.8)	4.2 (3.6-4.8)	0.0 (-0.1 to 0.1)	1.0
Arousal	4.2 (2.4-5.1)	4.2 (2.4-5.1)	0.0 (-0.2 to 0.2)	1.0
Lubrication	3.3 (2.7-3.6)	3.3 (2.7-3.6)	0.0 (-0.1 to 0.1)	1.0
Orgasm	3.6 (2.8-4.4)	3.6 (2.8-4.0)	0.0 (-0.1 to 0.1)	1.0
Satisfaction	4.4 (2.4-5.2)	4.0 (2.5-5.2)	-0.4 (-0.6 to -0.2)	0.001
Pain	3.6 (1.6-5.2)	3.2 (1.6-5.2)	-0.4 (-0.8 to 0.0)	0.030

Data are median (IQR). FSFI=Female Sexual Function Index, CI=confidence interval. *The FSFI is a validated 19-item index assessing 6 domains of sexual function: desire, arousal, lubrication, orgasm, satisfaction, and pain, on a scale of 1.2 – 6.0 (desire) or 0-6 (other domains). Higher scores indicate better function. †Data available for 758 and 755 women, respectively. ‡Adjusted for moment of CS. §The full scale score is the sum of the domain scores at each time point, ranges from 1.2-36. Effect estimates are calculated with single-layer as the reference group.

Table S7. Planned subgroup analyses regarding the primary outcome for single- versus double-layer closure

Subgroup	Single-layer (*n*=774)	Double-layer (*n*=770)	Subgroup specific adjusted median difference between groups (95% CI)*	Between-subgroup difference in adjusted median difference (95% CI)*	p value
**Moment of CS†**				0.0 (-0.17 to 0.17)	1.0
Prelabour	0.0 (0.0-2.0)	0.0 (0.0-2.0)	0.0 (-0.20 to 0.20)		
Intrapartum CS	0.0 (0.0-1.0)	0.0 (0.0-1.0)	0.0 (-0.16 to 0.16)		
**Emergency CS vs no emergency CS†**				0.0 (-0.14 to 0.14)	1.0
No emergency CS	0.0 (0.0-2.0)	0.0 (0.0-2.0)	0.0 (-1.13 to 1.13)		
Emergency CS	0.0 (0.0-2.0)	0.0 (0.0-1.0)	0.0 (-0.08 to 0.08)		
**Gestational age at time of CS**				0.0 (-0.40 to 0.40)	1.0
<37 weeks	0.0 (0.0-2.0)	0.0 (0.0-1.0)	0.0 (-0.74 to 0.74)		
≥37 weeks	0.0 (0.0-2.0)	0.0 (0.0-2.0)	0.0 (-0.16 to 0.16)		
**Dilatation†**				0.0 (-0.17 to 0.17)	1.0
≤3cm	0.0 (0.0-2.0)	0.0 (0.0-1.0)	0.0 (-0.20 to 0.20)		
>3cm	0.0 (0.0-1.0)	0.0 (0.0-1.0)	0.0 (-0.17 to 0.17)		
**Presence of placenta praevia**				0.0 (-0.14 to 0.14)	1.0
No placenta praevia	0.0 (0.0-1.8)	0.0 (0.0-1.0)	0.0 (-1.57 to 1.57)		
Placenta praevia	0.0 (0.0-3.0)	0.0 (0.0-2.0)	0.0 (-0.08 to 0.08)		
**Maternal comorbidity‡**				0.0 (-0.16 to 0.16)	1.0
Absent	0.0 (0.0-2.0)	0.0 (0.0-2.0)	0.0 (-0.18 to 0.18)		
Present	0.0 (0.0-2.0)	0.0 (0.0-1.0)	0.0 (-0.23 to 0.23)		
**Multiplicity**				0.0 (-0.14 to 0.14)	1.0
Singleton pregnancy	0.0 (0.0-2.0)	0.0 (0.0-1.0)	0.0 (-0.14 to 0.14)		
Twin pregnancy	0.0 (0.0-2.3)	0.0 (0.0-1.0)	0.0 (-0.69 to 0.69)		
**Type of menstruation†**				0.0 (-0.19 to 0.19)	1.0
Hormonally induced withdrawal bleeding	0.0 (0.0-1.0)	0.0 (0.0-0.0)	0.0 (-0.11 to 0.11)		
Natural cycle	0.0 (0.0-1.0)	0.0 (0.0-1.0)	0.0 (-0.29 to 0.29)		

Scores are reported as median (IQR). Analyses were performed using quantile regression. *Adjusted for moment of CS. †Unadjusted results are presented due to too complex quantile regression models after adjustment. ‡Including diabetes, hypertension or pre-eclampsia. P-value is the p-value of interaction term (treatment arm * subgroup).

Table S8. Clinical and sonographic outcomes within single-layer closure group, with or without application of endometrial saving technique

	**Endometrium included (n=743)**	**Endometrium excluded (n=293)**	**Adjusted effect estimate (95% CI)***	**p value**
**Clinical outcomes†**				
Spotting, days/month	0.0 (0.0-2.0)	0.0 (0.0-2.0)	Median difference 0.0 (-0.3 to 0.3)	1.0
Dysmenorrhoea, scale 0-10	4.0 (2.0-6.0)	4.0 (2.0-7.0)	Median difference 0.0 (-0.7 to 0.7)	1.0
**Sonographic outcomes available**	**656 (88.3%)**	**253 (86.3%)**	**—**	**—**
Niche prevalence	471 (71.8%)	150 (59.3%)	RR 0.83 (0.74 to 0.93)<br>RD -0.13 (-0.20 to -0.06)	0.001<br>0.000
RMT, N	635	242	—	—
RMT, mm	6.3 (3.1)	6.7 (3.8)	Mean difference 0.4 (-0.1 to 0.9)	0.082
Large niche prevalence (RMT ≤ 3mm)	90 (13.8%)	30 (11.9%)	RR 0.87 (0.59 to 1.27)<br>RD -0.03 (-0.07 to 0.02)	0.455<br>0.243
Large niche prevalence (RMT/AMT ratio < 50%)	232 (35.7%)	85 (33.9%)	RR 0.95 (0.78 to 1.16)<br>RD -0.02 (-0.09 to 0.05)	0.611<br>0.567
RMT/AMT ratio, N	627	238	—	—
RMT/AMT ratio	0.56 (0.41-0.74)	0.58 (0.41-0.81)	Median difference 0.01 (-0.04 to 0.06)	0.744

Data are median (IQR), number (%), or mean (SD) unless otherwise indicated. N represents the number of women with data available. CI=confidence interval, RMT=residual myometrium thickness, RR=relative risk, RD=risk difference, AMT=adjacent myometrium thickness. *Adjusted for moment of CS. †Available for 511 women in endometrium included group and 197 in endometrium excluded group. Effect estimates are calculated with endometrium included as the reference group.

Table S9. Per protocol analyses

	Single-layer (*n*=1131)	Double-layer (*n*=1112)	Adjusted effect estimate (95% CI)*	p value
**Menstrual outcomes**				
Spotting, days/month†	0.0 (0.0-2.0)	0.0 (0.0–1.0)	Median difference 0.0 (-0.1 to 0.1)	1.0
Presence of spotting†	270/766 (35.2%)	258/746 (34.6%)	RR 0.98 (0.85 to 1.12)<br>RD -0.01 (-0.06 to 0.04)	0.740<br>0.770
Presence of spotting ≥ 2 days/month†	195/766 (25.5%)	185/746 (24.8%)	RR 0.97 (0.82 to 1.15)<br>RD -0.01 (-0.05 to 0.04)	0.730<br>0.761
Total days blood loss, days/month†	6.0 (3.5)	5.9 (2.9)	Mean difference -0.1 (-0.4 to 0.3)	0.669
Duration of menstruation, days/month†	5.5 (2.3)	5.7 (2.5)	Mean difference 0.1 (-0.1 to 0.4)	0.280
Dysmenorrhoea, scale 0-10†	4.0 (2.0-6.0)	4.0 (2.0-6.0)	Median difference 0.0 (-0.5 to 0.5)	1.0
Need for treatment of gynaecological complaints‡	13/905 (1.4%)	25/876 (2.9%)	RR 1.97 (1.01 to 3.82)<br>RD 0.01 (-0.0 to 0.03)	0.045<br>0.086
**Perioperative outcomes**				
Operative time, N	1108	1090	—	—
Operative time, minutes	38.7 (11.5)	42.8 (11.1)	Mean difference 4.1 (3.2 to 5.1)	<0.001
Blood loss (mL), geometric mean (95% CI)	404 (390 to 418)	411 (396 to 426)	Geometric mean ratio 1.02 (0.97 to 1.07)	0.433
Need for additional hemostatic sutures	455 (40.3%)	446 (40.3%)	RR 1.00 (0.90 to 1.11)<br>RD 0.0 (-0.4 to 0.4)	0.987<br>0.985
Number of additional hemostatic sutures needed§	1 (1-2)	1 (1-2)	Median difference 0.0 (-0.2 to 0.2)	1.0
Hospital stay, N	1000	980	—	—
Hospital stay, days	3.0 (2.0 – 4.0)	3.0 (2.0 – 3.0)	Median difference 0.0 (-0.1 to 0.1)	1.0
Complication rate¶	114 (10.1%)	97 (8.7%)	RR 0.87 (0.67 to 1.13)<br>RD -0.01 (-0.04 to 0.01)	0.291<br>0.260
Readmission rate	13/987 (1.3%)	12/968 (1.2%)	RR 0.94 (0.43 to 2.06)<br>RD 0.00 (-0.01 to 0.01)	0.883<br>0.924
Missing, n	144	144	—	—
**Sonographic outcomes available**	984 (87.0%)	942 (84.7%)	—	—
Niche prevalence	679 (69.0%)	696 (73.9%)	RR 1.07 (1.01 to 1.13)<br>RD 0.05 (0.01 to 0.09)	0.025<br>0.021
RMT, N	949	903	—	—
RMT, mm	6.4 (3.3)	6.6 (3.4)	Mean difference 0.2 (-0.1 to 0.5)	0.148
RMT/AMT ratio	0.56 (0.41-0.75)	0.58 (0.41-0.74)	Median difference 0.01 (-0.02 to 0.04)	0.526
Large niche prevalence (RMT ≤ 3mm)	131 (13.4%)	111 (11.9%)	RR 0.89 (0.70 to 1.12)<br>RD -0.01 (-0.04 to 0.02)	0.323<br>0.679

Large niche prevalence (RMT/AMT ratio < 50%)	349 (35.8%)	331 (35.4%)	RR 0.99 (0.88 to 1.12)<br>RD -0.00 (-0.04 to 0.04)	0.879<br>0.961
Niche measurements**	—	—	—	—
Depth (mm), geometric mean (95% CI)	3.94 (4.07 to 3.82)	3.95 (3.83 to 4.07)	Geometric mean ratio 1.00 (0.96 to 1.04)	0.965
Length (mm), geometric mean (95% CI)	4.73 (4.54 to 4.92)	4.94 (4.76 to 5.13)	Geometric mean ratio 1.05 (0.99 to 1.11)	0.080
Width (mm), geometric mean (95% CI)	5.05 (4.84 to 5.27)	5.07 (4.87 to 5.28)	Geometric mean ratio 1.01 (0.95 to 1.07)	0.790
Niche volume†† (mm^3), geometric mean (95% CI)¶	23.0 (20.9 to 25.3)	25.2 (23.1 to 27.6)	Geometric mean ratio 1.10 (0.97 to 1.25)	0.144
Presence of intracavitary fluid‡‡	140 (14.3%)	153 (16.3%)	RR 1.14 (0.92 to 1.40)<br>RD 0.02 (-0.01 to 0.05)	0.233<br>0.266
Position uterus	—	—	—	—
Anteverted	690 (70.5%)	669 (71.6%)	Reference category	—
Stretched	64 (6.5%)	60 (6.4%)	RR 0.99 (0.70 to 1.39)<br>RD -0.00 (-0.02 to 0.02)	0.931<br>0.883
Retroverted	191 (19.5%)	174 (18.6%)	RR 0.96 (0.80 to 1.15)<br>RD -0.01 (-0.04 to 0.03)	0.632<br>0.629
Extremely retroverted (angle <45°)	34 (3.5%)	31 (3.3%)	RR 0.97 (0.60 to 1.56)<br>RD -0.00 (-0.02 to 0.02)	0.893<br>0.934
**PROMIS-APS outcomes§§**	52.7 (48.0-60.2)	52.7 (47.0-60.2)	Median difference 0.0 (-1.0 to 1.0)	1.0
**FSFI outcomes¶¶**	—	—	—	—
Desire	4.2 (3.6-4.8)	4.2 (3.6-4.8)	Median difference 0.0 (-0.1 to 0.1)	1.0
Arousal	4.2 (2.4-5.1)	4.2 (2.4-5.1)	Median difference 0.0 (-0.2 to 0.2)	1.0
Lubrication	3.3 (2.7-3.6)	3.3 (2.7-3.6)	Median difference 0.0 (-0.1 to 0.1)	1.0
Orgasm	3.6 (2.8-4.4)	3.6 (2.8-4.0)	Median difference 0.0 (-0.1 to 0.1)	1.0
Satisfaction	4.4 (2.4-5.2)	4.4 (2.4-5.2)	Median difference -0.4 (-0.6 to -0.2)	0.001
Pain	3.6 (1.6-5.2)	3.2 (1.6-5.2)	Median difference -0.4 (-0.8 to -0.0)	0.034
Full scale***	23.2 (19.5-25.9)	23.0 (19.0-25.4)	Median difference -0.3 (-0.9 to 0.3)	0.341

Data are median (IQR), number (%), or mean (SD), unless otherwise indicated; N represents the number of women with data available. CI=confidence interval, RR=relative risk, RD=risk difference, RMT=residual myometrium thickness, AMT=adjacent myometrial thickness, PROMIS-APS=PROMIS Ability to Participate in Social Roles and Activities v2.0 short form 8a, FSF=Female Sexual Function Index. *Adjusted for moment of CS. †Data available for 766 women in single-layer group and 746 women in double-layer group. ‡Data available for 905 women in single-layer group and 876 women in double-layer group. §Only recorded when at least one additional hemostatic suture was needed. ¶Fever, bladder/intestinal lesion, postpartum haemorrhage or other. **Only recorded when a niche was present. ††Calculated as $1/3 * \pi * (1/2 * \text{length})^2 * \text{depth}$. ‡‡Only available from transvaginal ultrasound, not when contrast was used. §§Data available for 956 women in single-layer group and 940 in double-layer group; T-score that represents a standardised score with a mean of 50 and SD of 10. ¶¶Data available for 752 women in single-layer group and 733 women in double-layer group; reported on a scale of 1.2-6.0 (desire) or 0.0-6.0 (other domains).***Sum of domain scores, range from 1.2-36.0. Effect estimates are calculated with single-layer as the reference group.

Table S10. Per protocol analyses SF-36 outcomes

SF-36 domain	Measurement moment	Single-layer (n=1131)*	Double-layer (n=1112)*	p-value for comparison of change between study arms
	3 months	52.4 (7.6)	52.1 (7.8)	—
PCS	9 months	54.3 (7.3)	53.6 (7.9)	—
	Change	1.9 (6.1)	1.6 (6.6)	0.446†
	3 months	50.1 (9.9)	50.2 (9.1)	—
MCS	9 months	49.3 (9.5)	48.6 (10.1)	—
	Change	-1.1 (8.8)	-1.6 (8.9)	0.208†
	3 months	95 (85-100)	95 (85-100)	—
Physical functioning	9 months	100 (90-100)	95 (90-100)	—
	Change	5 (0-10)	0 (0-10)	0.349‡
	3 months	100 (50-100)	100 (50-100)	—
Physical role	9 months	100 (100-100)	100 (75-100)	—
	Change	0 (0-25)	0 (0-25)	0.249‡
	3 months	89.8 (67.4-100)	89.8 (67.4-100)	—
Bodily pain	9 months	89.8 (77.6-100)	89.8 (69.4-100)	—
	Change	0 (0-12.2)	0 (-2 - 12.2)	0.743‡
	3 months	75 (65-90)	75 (65-90)	—
General health	9 months	75 (60-85)	70 (55-85)	—
	Change	0 (-10 - 5)	-5 (-15 - 5)	0.042‡
	3 months	100 (66.7-100)	100 (100-100)	—
Emotional role	9 months	100 (100-100)	100 (66.7-100)	—
	Change	0 (0-0)	0 (0-0)	0.172‡
	3 months	60 (45-75)	60 (50-70)	—
Energy/vitality	9 months	60 (45-70)	60 (45-70)	—
	Change	0 (-10 - 10)	0 (-10 - 10)	0.663‡
	3 months	84 (72-92)	84 (72-92)	—
Mental health	9 months	80 (72-88)	80 (68-88)	—
	Change	0 (-8 - 4)	0 (-8 - 4)	0.945‡
	3 months	87.5 (62.5-100)	87.5 (62.5-100)	—
Social functioning	9 months	87.5 (75-100)	87.5 (75-100)	—
	Change	0 (0-12.5)	0 (-12.5 - 12.5)	0.018‡

Scores reported as mean (SD), or median (IQR). The Medical Outcomes Study 36-Item Short Form Health Survey (SF-36) general health-related quality of life scores range from 0 (severely affected) to 100 (not affected). CI=confidence interval, PCS=physical component summary score, MCS=mental component summary score. Change= 9 months score minus 3 months score. Both summary scores are z-scores compared with Dutch female reference population with mean of 50, standard deviation (SD) of 10. *3 months score available for 961 and 941 women, respectively; 9 months score available

for 875 and 846 women, respectively; change score available for 832 and 807 women, respectively. †p-value for interaction term of treatment arm and measurement moment in linear mixed model; ‡Mann-Whitney test on change scores.

Table S11. Differences in baseline characteristics between responders and non-responders for the primary outcome

Variables	9 month follow-up unavailable (n=416)	9 month follow-up available (n=1876)	p-value
<u>**Patient-reported***</u>			
Age, years	31.3 (5.0)	32.1 (4.6)	0.014
Body-mass index (kg/m^2)	26.3 (4.7)	26.5 (4.7)	0.426
**Continent of origin**			0.000
Europe	216 (88.9%)	1695 (95.0%)	
Asia	15 (6.2%)	28 (2.1%)	
Middle- or South America	7 (2.9%)	23 (1.3%)	
Africa	4 (1.6%)	9 (0.5%)	
Northern America	1 (0.4%)	18 (1.0%)	
Other	0 (0.0%)	2 (0.1%)	
Current smoker	21 (8.6%)	89 (5.0%)	0.018
**Level of education**			0.000
Low	26 (10.7%)	112 (6.3%)	
Middle	103 (42.4%)	575 (32.2%)	
High	111 (45.7%)	1086 (60.8%)	
Other	3 (1.2%)	12 (0.7%)	
Nulliparous	171 (71.0%)	1357 (77.0%)	0.038
Missing, n			
Previous miscarriage or abortion	92 (37.9%)	556 (31.1%)	0.035
Underwent curettage, n/N (%)	54/92 (59.3%)	260/556 (46.8%)	0.026
Previous ectopic pregnancy	4 (1.6%)	27 (1.5%)	0.874
Gestational age at CS (weeks)	38.9 (37.6 – 39.4)	39.0 (38.0 – 39.7)	0.005
Preterm delivery <37 weeks	48 (19.8%)	227 (12.7%)	0.003
Hypertensive disorder†	47 (19.3%)	318 (17.9%)	0.583
Diabetes (mellitus or gestational)	27 (11.2%)	176 (9.9%)	0.518
<u>**Characteristics from hospital file**</u>			
Twin pregnancy	45 (10.8%)	126 (6.7%)	0.004
**Prelabour caesarean section, reason**	227 (54.6%)	1172 (62.5%)	0.003
Breech presentation, n/N (%)	112/227 (49.3%)	656/1172 (56.0%)	
Placenta praevia, n/N (%)	21/227 (9.3%)	97/1172 (8.3%)	
Traumatic vaginal delivery in the past, n/N (%)	26/227 (11.5%)	95/1172 (8.1%)	0.212
Twin pregnancy, n/N (%)	17/227 (7.5%)	62/1172 (5.3%)	
Other, n/N (%)	51/227 (22.5%)	262/1172 (22.4%)	
**Intrapartum caesarean section, reason**	189 (45.4%)	704 (37.5%)	0.003
Failure to progress in 1st stage, n/N (%)	117 (61.9%)	395 (56.1%)	
Failure to progress in 2nd stage, n/N (%)	28 (14.8%)	138 (19.6%)	
Fetal compromise, n/N (%)	27 (14.3%)	100 (14.2%)	0.438
Failed induction, n/N (%)	5 (2.6%)	30 (4.3%)	
Other, n/N (%)	12 (6.3%)	41 (5.8%)	
Induction of labour	121 (29.1%)	457 (24.4%)	0.045
Received augmentation with oxytocin	162 (38.9%)	608 (32.4)	0.011
Contractions present	217 (52.2%)	819 (43.7%)	0.002
Dilatation present	203 (48.8%)	770 (41.0%)	0.007

Missing, n	89	516	
**Dilatation cm**	5 (4-8)	6 (4-9)	0.016
≤3 cm, n/N (%)	44/203 (21.7%)	136/768 (17.7%)	
4-7 cm, n/N (%)	103/203 (50.7%)	362/768 (47.1%)	0.103
≥8 cm, n/N (%)	56/203 (27.6%)	270/768 (35.2%)	
Missing	0	2	
**Fetal station at moment of decision**			
Elective CS, station unknown, n/N (%)	187 (49.9%)	978 (54.9%)	
Hodge 0-1, n/N (%)	141 (37.6%)	575 (32.3%)	
Hodge 2, n/N (%)	39 (10.4%)	195 (10.9%)	0.235
Hodge 3-4, n/N (%)	8 (2.1%)	34 (1.9%)	
Emergency caesarean section‡	33 (7.9%)	137 (7.3%)	0.657

Data are mean (±SD), n (%), or median (IQR), unless otherwise indicated; N is equal to the total number of patients in the group, unless otherwise indicated; CS=caesarean section.

*Data available for 1013 (88.5%) women from single-layer group and 1015 (88.4%) of women from double-layer group. †Defined as pregnancy induced hypertension or pre-eclampsia/hemolysis; elevated liver enzymes; low platelets (HELLP) syndrome. ‡Defined as severe fetal distress or maternal disease and necessity for immediate delivery within several minutes.

References

1. Aaronson NK, Muller M, Cohen PD, et al. Translation, validation, and norming of the Dutch language version of the SF-36 Health Survey in community and chronic disease populations. *J Clin Epidemiol* 1998; **51**(11): 1055-68.
2. Dolan P. Modeling valuations for EuroQol health states. *Med Care* 1997; **35**(11): 1095-108.
3. Rosen R, Brown C, Heiman J, et al. The Female Sexual Function Index (FSFI): a multidimensional self-report instrument for the assessment of female sexual function. *J Sex Marital Ther* 2000; **26**(2): 191-208.
4. PROMIS. Ability to Participate in Social Roles and Activities, Short Form 8a. 2017. http://www.healthmeasures.net/explore-measurement-systems/promis/intro-to-promis/list-of-adult-measures.
