# Supplementary material for: Effect of single‐ versus double‐layer uterine closure during caesarean section on postmenstrual spotting (2Close): multicentre, double‐blind, randomised controlled superiority trial
Source: BJOG. 2020 Oct 25;128(5):866–78. doi: 10.1111/1471-0528.16472 (PMC7983985; doi:10.1111/1471-0528.16472)
Supplement: Supplementary file 3 — Appendix S1. Further discussion. [file BJO-128-866-s013.pdf]

Appendix S1. Further discussion

Screening eligible women

Screening was performed on the outpatient clinic from Monday to Friday, and on the labor ward every day Patients from all providers were available for screening and enrollment in the study. In the Netherlands, CS is only carried out in hospitals and not in (private) clinics. Referral to the hospital for consultation takes place during pregnancy and labor from midwifery practices. We observed similar proportions of pre-labor and during labor CS in the majority of hospitals. In practice, we experienced that it was easier to recruit and inform eligible patients a few weeks before elective CS at the outpatient clinic compared to counseling during labor. This resulted in an overrepresentation of women with elective CS.

Differences between responders and non-responders for the primary outcome

We observed two large differences between responders (n=1876) and non-responders (n=416) to our nine month questionnaire to determine the primary outcome in Table S11. Non-responders had, in general, a lower socio-economic status based on educational level, smoking habits, and had more often a foreign background. This could have led to less loyalty regarding responding to the digital questionnaires. Second, non-responders had more often an in labor CS. It could well be that they, compared to pre-labor CS, had or took less time to read the patient information which included a paragraph about digital questionnaires and the need to answer our questions.

These subanalyses suggest that our responders have relative low risk on development of a symptomatic niche, since more planned CS were performed in this group which is a protective factor for niche development (dilatation and induction are risk factors.[1-3] This means that the 83% responders show an underestimation of niche related spotting than in daily practice. Extrapolation of these results is therefore more difficult due to differences in baseline characteristics and known risk factors between responders and non-responders.

Interpretation [in the light of other evidence]

We have probably overestimated the number of days of postmenstrual spotting when calculating the sample size, as scarcity of literature forced us to rely on data from studies with a selected symptomatic population.[4,5] In the present study, we reported a median number of days of postmenstrual spotting due to the skewed distribution of this outcome. It might be difficult to interpret a median number of days of spotting of 0 in both groups, since it is dominated by the 65% of women experiencing no spotting. We have shown, however, that spotting is a relevant problem after CS since 35% of the participants experienced at least one day of spotting per month with a mean of 3.7 days in this group.

References:

1. Bij de Vaate AJ, van der Voet LF, Naji O, et al. Prevalence, potential risk factors for development and symptoms related to the presence of uterine niches following Cesarean section: systematic review. *Ultrasound Obstet Gynecol* 2014; **43**(4): 372-82.
2. Voet L, Vaate A, Heymans MW, Brolmann HAM, Veersema S, Huirne JAF. Prognostic Factors for Niche Development in the Uterine Caesarean Section Scar. *Eur J Obstet Gynecol Reprod Biol* 2017; **213**: 31-2.
3. Vikhareva Osser O, Valentin L. Risk factors for incomplete healing of the uterine incision after caesarean section. *BJOG* 2010; **117**(9): 1119-26.
4. Vervoort AJMW, van der Voet LF, Hehenkamp WJK, et al. Hysteroscopic resection of a uterine caesarean scar defect (niche) in women with postmenstrual spotting: a randomised controlled trial. *BJOG* 2018; **125**(3): 326-34.

5. Vervoort AJMW, Vissers J, Hehenkamp WJK, Brölmann HAM, Huirne JAF. The effect of laparoscopic resection of large niches in the uterine caesarean scar on symptoms, ultrasound findings and quality of life: a prospective cohort study. *BJOG* 2018; **125**(3): 317-25.
